# Bioinformatic Analysis of *Bacillus pacificus* B630: Molecular Understanding of Biofilm Production

**DOI:** 10.3390/ijms27177743

**Published:** 2026-08-29

**Authors:** Luis-Daniel Sánchez-Arcos, José-Humberto Pérez-Olais, Alberto Patricio-Hernández, Hugo-Alberto Rodríguez-Ruiz, Verónica-Iranzú Martínez-Santos, Yuridia Mercado-Flores, Karen Cortés-Sarabia, Salvador Muñoz-Barrios, Arturo Ramírez-Peralta

**Affiliations:** 1 Laboratorio de Investigación en Patometabolismo Microbiano, Facultad de Ciencias Químico-Biológicas, Universidad Autónoma de Guerrero, Av. Lázaro Cárdenas S/N, Ciudad Universitaria, Colonia La Haciendita, Chilpancingo C.P. 39090, Mexico; DanielArc_Bio@outlook.com; 2 Unidad de Investigación en Virología y Cáncer, Hospital Infantil Federico Gómez, Dr. Márquez 162, Colonia Doctores, Alcaldía Cuauhtémoc, Ciudad de México C.P. 06720, Mexico; jholais@gmail.com; 3 Facultad de Ciencias Agropecuarias y Ambientales, Universidad Autónoma de Guerrero, Teloloapan S/N, Colonia Ignacio Manuel Altamirano, Iguala de la Independencia C.P. 40015, Mexico; patriciofcqb@gmail.com; 4 Laboratorio de Investigación en Obesidad y Diabetes, Facultad de Ciencias Químico-Biológicas, Universidad Autónoma de Guerrero, Av. Lázaro Cárdenas S/N, Ciudad Universitaria, Colonia La Haciendita, Chilpancingo C.P. 39090, Mexico; hugordzrz@outlook.com; 5 SECIHTI, Universidad Autónoma de Guerrero, Av. Javier Méndez Aponte 1, Fraccionamiento Servidor Agrario, Chilpancingo C.P. 39070, Mexico; iranzu23@gmail.com; 6 Laboratorio Nacional Conahcyt de Investigación y Servicios para la Productividad del Campo, Universidad Politécnica de Pachuca, Carretera Pachuca-Cd. Sahagún km 20, Rancho Luna, Ex Hacienda de Santa Bárbara, Zempoala C.P. 43830, Mexico; yuridiamercado@upp.edu.mx; 7 Laboratorio de Investigación en Inmunobiología y Diagnóstico Molecular, Facultad de Ciencias Químico-Biológicas, Universidad Autónoma de Guerrero, Av. Lázaro Cárdenas S/N, Ciudad Universitaria, Colonia La Haciendita, Chilpancingo C.P. 39090, Mexico; kcortes_sarabia@hotmail.com; 8 Laboratorio de Investigación en Inmunología y Microbiología, Facultad de Ciencias Naturales, Universidad Autónoma de Guerrero, Avenida Universidad S/N, Carr. Nal. Chilpancingo-Petaquillas, Ex Rancho Shalako, Las Petaquillas C.P. 39050, Mexico; smunoz@uagro.com

**Keywords:** *Bacillus cereus*, *Bacillus pacificus*, biofilm, bioinformatic analysis

## Abstract

The aim of this study was to determine biofilm production and motility in *Bacillus pacificus* B630 and *Bacillus cereus* ATCC 14579, and to perform a comparative genome analysis using bioinformatic tools to understand the differences between the two strains. Biofilm production was performed in glass tubes stained with safranin; motility was determined on soft agar. Bioinformatic analysis was performed using genomic information from both strains, including the identification of orthologous genes, the similarity between genes of the *eps1* and *sipW-tasA-calY* operons, and the SipW and TasA model prediction. *B. pacificus* B630 produces a greater amount of biofilm on glass than *B. cereus* ATCC 14579 (*p <* 0.01). Furthermore, *B. pacificus* B630 shows lower motility than *B. cereus* ATCC 14579 (*p <* 0.001). *B. pacificus* B630 contains 45 unshared genes, whereas *B. cereus* ATCC 14579 has 27 unshared genes. Differences in similarity were observed between the genes of the *eps1* and *sipW-tasA-calY* operons. These differences between SipW and TasA may affect protein structural predictions. In SipW, the differences may affect the C-terminal region. In TasA, the number of B-sheets differed between the two proteins, and amino acid substitutions were found in regions of high protein aggregation. Genomic differences in genes associated with biofilm production may explain differences in biofilm production between the strains studied.

## 1. Introduction

The *Bacillus cereus* group, also known as *B. cereus sensu lato* (*s.l.*), is a species complex assigned to the genus *Bacillus* and comprises Gram-positive, aerobic or facultatively anaerobic, endospore-forming bacteria with low GC content that belong to the phylum Firmicutes and display high genetic similarity [1,2]. Since the advent of whole-genome sequencing, the number of available genomes from species within the *B. cereus* group has allowed the inclusion of at least 21 species encompassed within a total of 12 genomospecies [1].

Despite this genetic similarity, genomic differences have enabled strain diversification within the group, which includes strains of clinical, economic, and food-related importance. *B. cereus sensu stricto* (*s.s.*) is recognized as a cause of foodborne intoxication, including emetic and diarrheal syndromes, severe non-gastrointestinal infections, and food spoilage. *B. anthracis* is the etiological agent of anthrax, and *B. thuringiensis* is used as a biopesticide [1,3,4,5,6]. In contrast, the group also contains beneficial strains, including plant growth-promoting and probiotic bacteria [5,7].

Strains of *B. cereus s.l.* are able to synthesize diverse virulence factors, including toxins, form resistant endospores, and produce biofilms [3]. Biofilms are considered a mechanism of environmental persistence [8]; moreover, in the food industry, biofilm formation has been considered a potential source of systemic contamination during food production [9].

Among strains of the *B. cereus* group, *B. pacificus* strains are capable of producing abundant biofilms. Hayrapetyan et al. (2015) [10] reported that *B. cereus* strain ATCC 10987 (currently reclassified as *B. pacificus* ATCC 10987 [11]) produces a greater amount of biofilm than *B. cereus* strain ATCC 14579. In our research group, a *B. pacificus* strain was isolated from rice samples that produced abundant biofilm on glass [12,13]; another study showed that *B. pacificus* strain B630 produces a greater amount of biofilm on eggshell than *B. cereus* strain ATCC 14579 [14], a behavior similar to that previously reported for strains of this group [10].

Therefore, the aim of this study was to determine biofilm production and motility in *B. pacificus* B630 and *B. cereus* ATCC 14579, and to perform a comparative genome analysis using bioinformatic tools in order to understand the differences between the two strains.

## 2. Results and Discussion

### 2.1. Biofilm Production and Motility Assay

**Figure 1:**
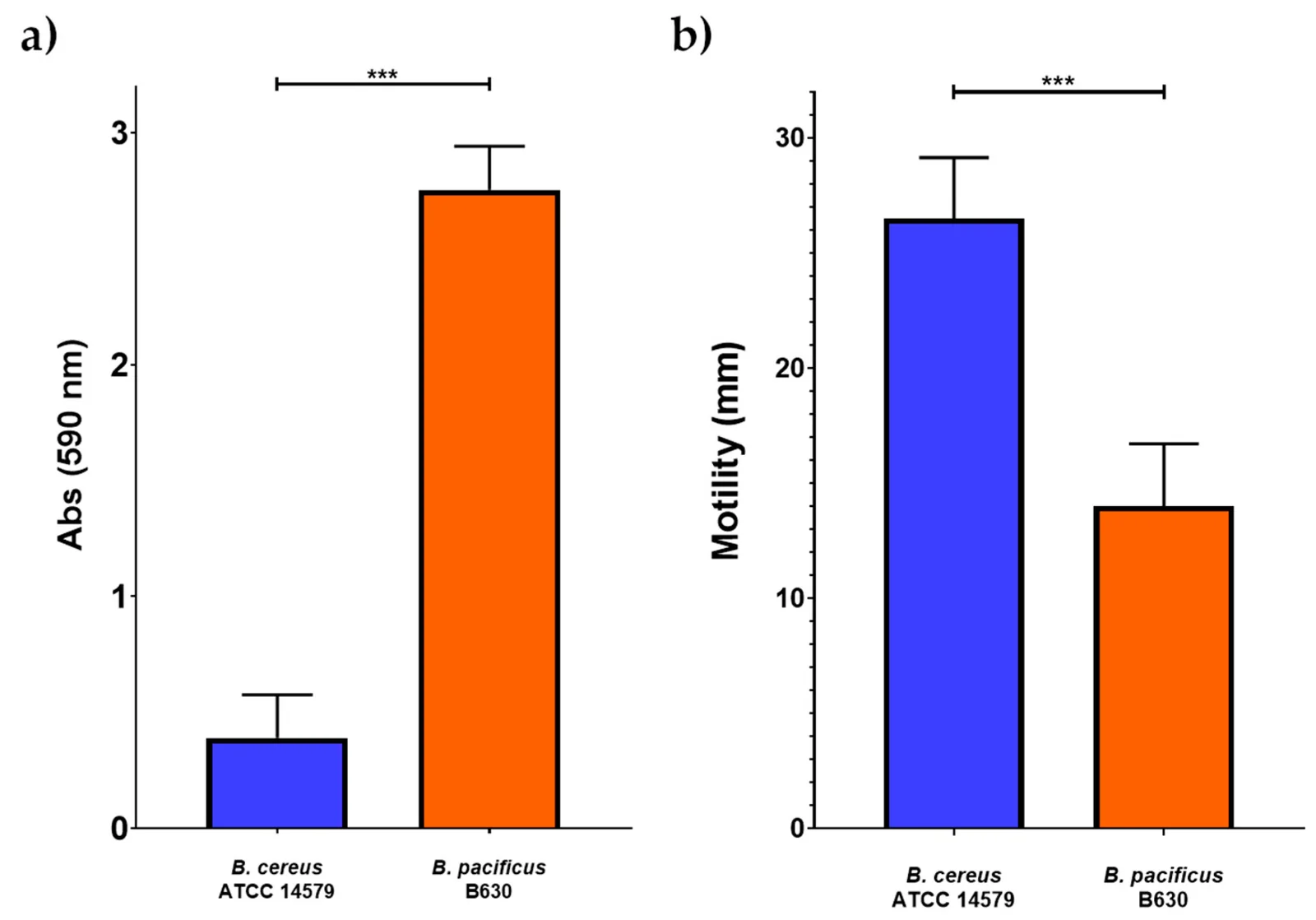
Biofilm production and motility in the study strains. (**a**) Biofilm production. (**b**) Motility assay. Statistical analysis was performed using *t*-test, *p* < 0.001 ***.

*B. pacificus* strain B630 was isolated in a previous study from rice-based food products; this strain stood out for its ability to produce abundant biofilm on glass [12]. Whole-genome sequencing was subsequently undertaken in a follow-up study, given that only strains with low biofilm-forming capacity on glass had previously been reported within the research group [15,16,17,18,19]. Although the previous phylogenomic analysis classified it as *B. pacificus* [13], in this study it was decided to compare it with *B. cereus* ATCC 14579, considering the experimental data obtained, which demonstrate that *B. pacificus* B630 produces a greater amount of biofilm on glass than *B. cereus* ATCC 14579 (*p <* 0.001; Figure 1a). Furthermore, *B. pacificus* B630 shows lower motility than *B. cereus* ATCC 14579 (*p <* 0.001; Figure 1b).

### 2.2. Comparative Analysis of Orthologous Gene Families

**Figure 2:**
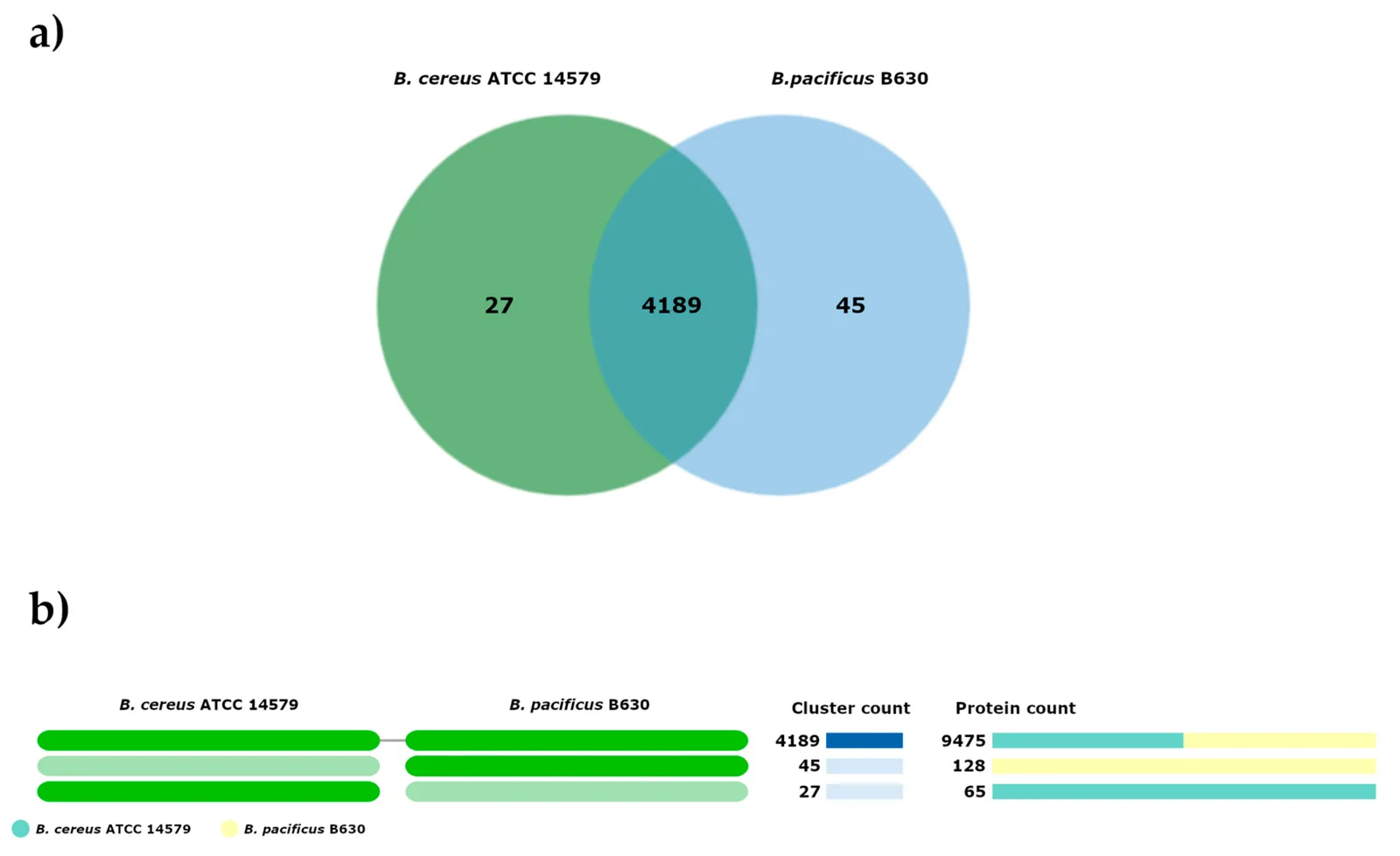
Representation of the OrthoVenn3 analysis results among *B. pacificus* B630 and *B. cereus* ATCC 14579. (**a**) Visual representation of cluster sharing and unique clusters between the strains. (**b**) Graphical visualization of orthology cluster analysis results among both strains and the number of proteins. In the left panel, green bar intensity indicates the number of proteins. In the center panel, blue intensity indicates the number of genes. In the right panel, yellow indicates proteins specific to *B. pacificus* B630, while aquamarine indicates those of *B. cereus* ATCC 14579.

Orthologous gene analysis revealed that both strains share 4189 genes; this is not unexpected, considering the definition of the *B. cereus s.l.* group as a group of strains with high genetic similarity [2,20]. However, *B. pacificus* B630 contains 45 unshared genes, whereas *B. cereus* ATCC 14579 has 27 unshared genes (Figure 2). Among the genes uniquely annotated in each strain, those related to cell wall organization and metabolic pathways (aromatic compound, DNA, RNA, and carbohydrate/polysaccharide metabolism) were found in *B. pacificus* B630, whereas genes related to metabolism (nitrogenous compound, alcohol, organic acid, and aromatic compound metabolism) and pathogenicity (siderophore and hemolysin production) were found in *B. cereus* ATCC 14579 (Tables S1–S4).

### 2.3. Genes Related to Biofilm Production

**Figure 3:**
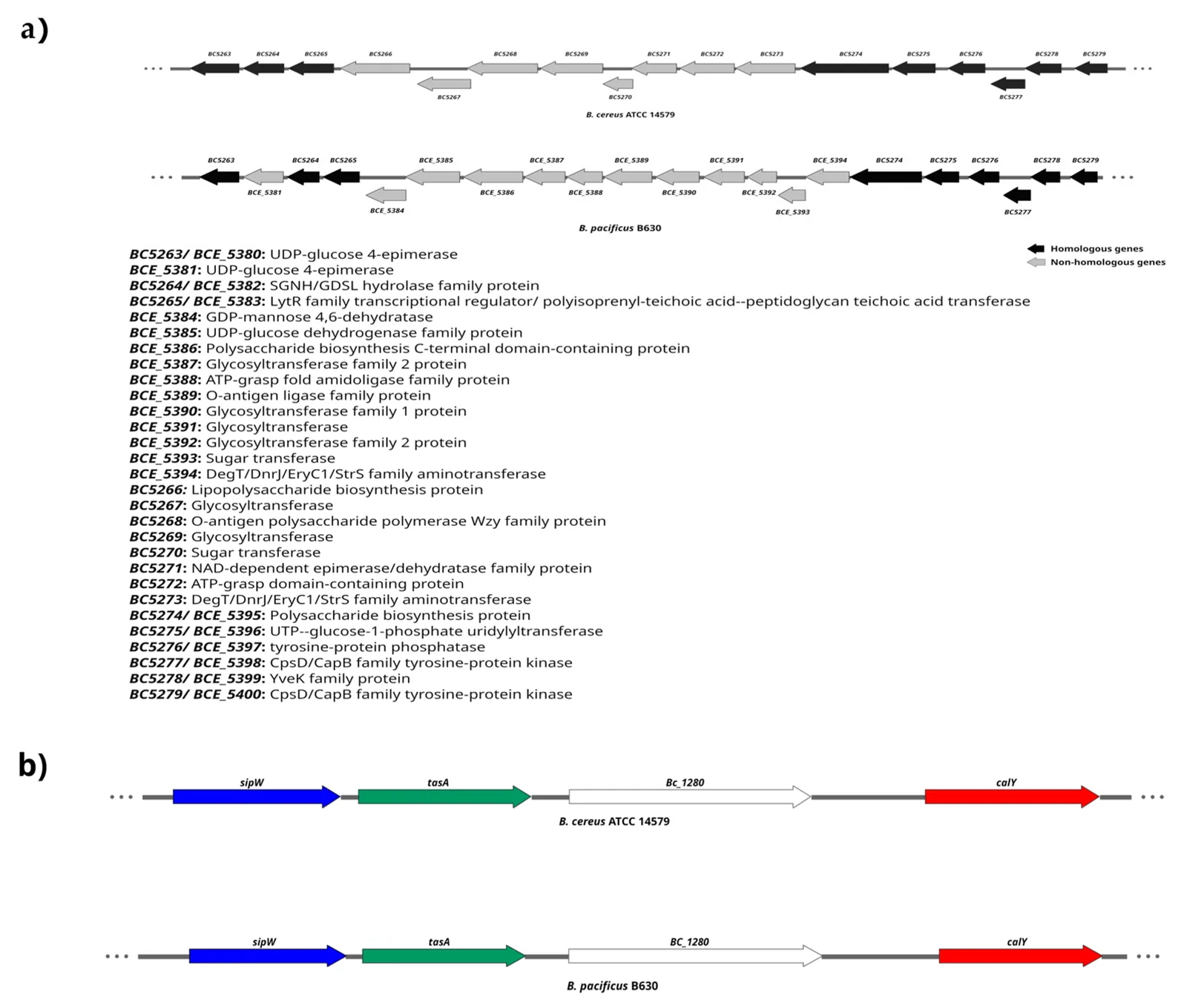
Comparison of the *eps1* and *sipW-tasA-calY* operons between *B. cereus* ATCC 14579 and *B. pacificus* B630. (**a**) Gene cluster of the *eps1* operon of *B. cereus* ATCC 14579 and its homologs in *B. pacificus* B630. (**b**) Organization of the *sipW*-*tasA*-*calY* operon in *B. cereus* ATCC 14579 and *B. pacificus* B630.

In the genomics of biofilm formation among *B. cereus s.l.* strains, several operons with specific biofilm-related functions have been described. The *eps1* region (genes *BC5263*–*BC5279*, homologous to the *epsA-O* operon of *Bacillus subtilis*) and the *eps2* region (genes *BC1583*–*BC1591*, annotated as involved in capsular polysaccharide biosynthesis) have been functionally characterized in *B. cereus* ATCC 14579 using deletion mutants. That study showed that *eps1* contributes to a form of social bacterial motility that requires functional flagella, whereas *eps2* is primarily involved in bacterial adhesion, cell-to-cell aggregation, biofilm formation, and host–cell interaction, and is dispensable for motility [21]. Based on the VFDB analysis, *B. pacificus* B630 was found to contain the capsular polysaccharide synthesis-related genes *BC5263*, *BC5264*, *BC5265*, *BC5274*, *BC5275*, *BC5276*, *BC5277*, *BC5278*, and *BC5279*, which are homologous to the genes present in *B. cereus* ATCC 14579 that form part of the *eps1* operon. Although both strains carry orthologous *eps1* operon genes, *B. pacificus* B630 exhibited lower motility than *B. cereus* ATCC 14579 (Figure 1b). It should be noted that expression levels of the *eps1* operon were not determined in this study; therefore, the extent to which this operon contributes to the observed motility phenotype cannot be established from sequence data alone. Two genomic features were nonetheless considered as possible contributing factors: first, nucleotide identity between the *eps1* orthologs ranged from 83.99% to 93.65% in *B. pacificus* B630 relative to *B. cereus* ATCC 14579 (Table 1); second, comparison of the genomic region spanning this operon revealed strain-specific genes that were not shared between the two strains (Figure 3a). These sequence-level differences may, in principle, be associated with the motility phenotype, although this remains to be confirmed through expression analysis (e.g., RT-qPCR) or functional assays in a future project.

The *sipW*-*tasA*-*calY* operon contains genes related to the formation of amyloid-type fibers characteristic of air–liquid interface biofilms, attributed to the amyloid-type protein TasA and its processing by SipW [22]. This same operon also encodes the CalY protein, which has two functions during biofilm formation: in the early stages it acts as an adhesin, and later it becomes part of the fibrillar matrix of mature biofilms [23]; its role during bacterial autoaggregation has also been described [21]. In this study, we identified all genes within the *sipW-tasA-calY* region in both strains analyzed. The genomic organization of this region had previously been described in another *B. cereus* strain (*B. cereus* CECT148) using the genome of the *B. cereus* ATCC14579 strain as a reference [22], and the findings align with those reported in the present study (Figure 3b). Regarding nucleotide similarity, the *sipW*, *tasA*, and *calY* genes of *B. pacificus* B630 show percentages of 89.14%, 78.43%, and 84.51%, respectively, relative to the corresponding genes of *B. cereus* ATCC 14579 (Table 1).

Furthermore, a phylogenetic analysis of the nucleotide sequences of the *eps1* and *sipW-tasA-calY* regions from 21 strains—including *B. cereus* ATCC 14579 and *B. pacificus* B630 and encompassing various species of the *B. cereus s.l.* group—was conducted; this revealed that nucleotide conservation in the *sipW-tasA-calY* region is maintained at the species level. In *B. pacificus B630*, this genomic region clusters with other *B. pacificus* strains (Figures S1 and S2).

### 2.4. Topology, Modeling, Comparative Analysis, and Structural Validation of the SipW Protein

The TasA protein and its processing by SipW, as well as their relationship with biofilm production in *B. subtilis* and *B. cereus*, have been characterized functionally, structurally, and biochemically in several studies [24,25,26,27,28,29,30,31,32,33,34]. Accordingly, this study proceeded with a more in-depth genetic analysis and structural modeling of the SipW and TasA proteins.

**Figure 4:**
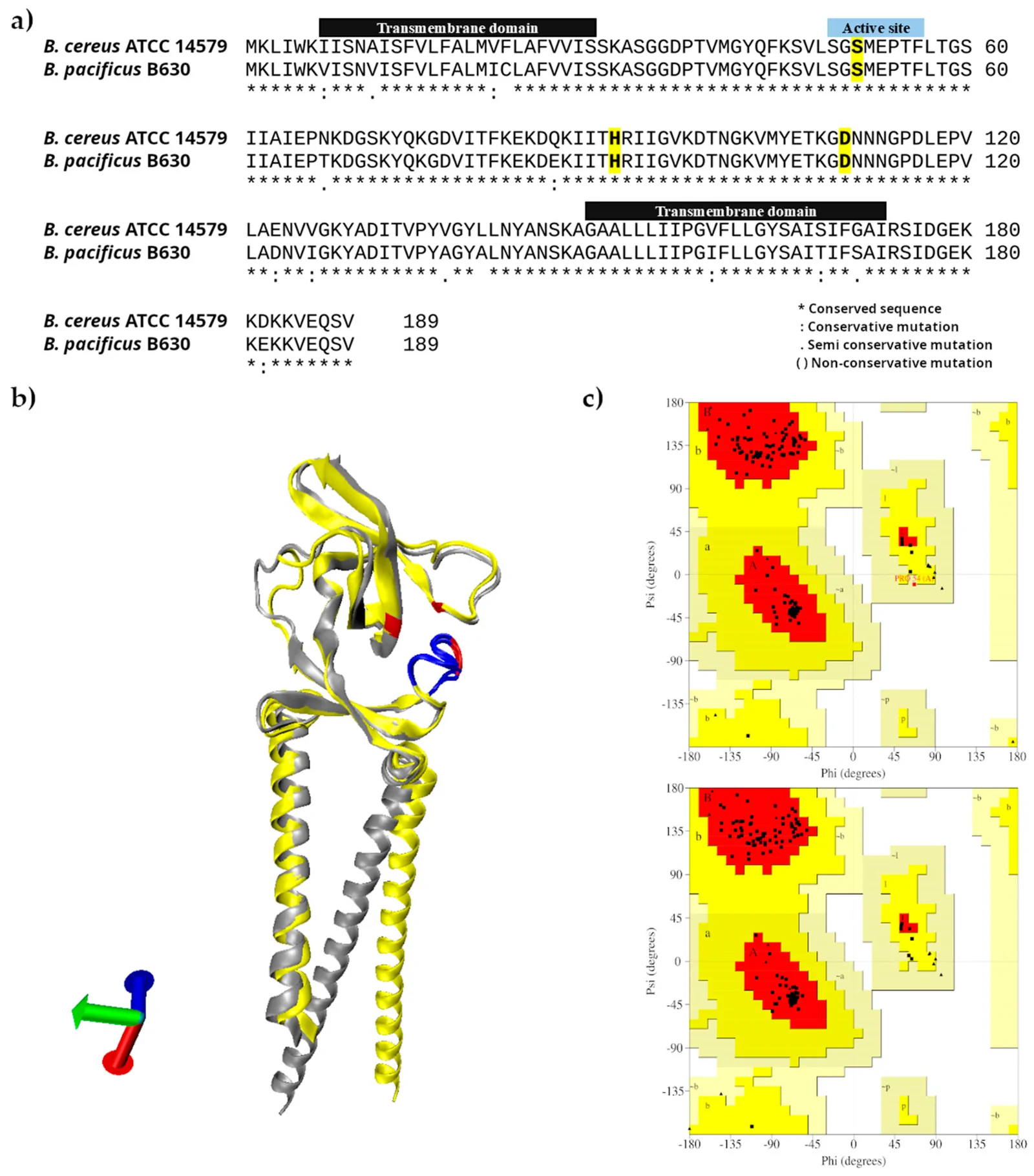
SipW protein of both study strains. (**a**) Amino acid sequence analysis of SipW from *B. pacificus* B630 compared with *B. cereus* ATCC 14579. Black boxes indicate the location of transmembrane domains; blue box indicates the active site, and yellow marks indicates key amino acids of the SipW protein. (**b**) AlphaFold3 prediction of the SipW proteins of *B. cereus* ATCC 14579 and *B. pacificus* B630. The yellow model corresponds to *B. pacificus* B630, while the silver model corresponds to *B. cereus* ATCC 14579. The blue region indicates the active site and the red region the key amino acids. Editing was performed in Visual Molecular Dynamics (VMD) v.1.9.4. (**c**) Validation model using SAVES (v.6.1) with PROCHECK. After refinement with GalaxyWEB, the Ramachandran plots show that 98.2% and 98.1% of the SipW amino acid residues of *B. cereus* ATCC 14579 and *B. pacificus* B630, respectively, are located in the most favored region. Confidence metrics obtained for the structural models of the SipW protein are included in Table S5.

In *B. subtilis*, SipW has been described as a member of the endoplasmic reticulum (ER) subfamily of type I signal peptidases and can process TasA into its mature form, which is exported to the extracellular matrix [32,33,35]. Caro-Astorga et al. (2014) [22] described orthologous *sipW* and *tasA* genes in *B. cereus* related to biofilm production. In this study, nucleotide identity analysis revealed differences in the *sipW* genes of *B. cereus* ATCC 14579 and *B. pacificus* B630, prompting an amino acid sequence analysis. Initial comparison with the orthologous *B. subtilis* gene identified serine-51, histidine-91, and aspartic acid-110 (Figure 4a), residues reported to be essential for peptidase activity [34]. Strains carrying individual mutations [*SipW(S47A)*, *SipW(H87A)*, and *SipW(D106A)*] do not lose their adhesion capacity, but the *SipW(S47A)* strain loses its ability to form floating biofilms [35]; this has been attributed to the fact that TasA, when not processed at its N-terminal region, remains anchored to the bacterial cell wall, thereby retaining its adhesive capacity but not its fiber-forming capacity [34,35].

Further analysis of both strains identified the presence of transmembrane domains (one located in the N-terminal domain and another in the C-terminal domain) and greater variability in the C-terminal region (Figure 4a). Identification of these transmembrane domains was also confirmed through analysis with Protter 1.0, and a cytoplasmic tail was detected in the C-terminal domain (Figure S3).

The endoplasmic reticulum signal peptidase subfamily contains a transmembrane domain and a highly conserved catalytic domain with a serine residue at the active site, whereas the C-terminal region contains a transmembrane domain, an extracellular loop, and a cytoplasmic tail, representing a region of greater variability [34]. This last point was also demonstrated in this study during structural modeling of this protein, in which a conformational change was predicted at the C-terminal position (Figure 4b); even when extending this structural analysis to a larger number of SipW sequences, this same variability was identified when comparing the SipW structure of *B. subtilis*, another *B. pacificus* strain (*B. pacificus* ATCC 10987), and the two study strains (Figure S4).

Terra et al. (2012) [35] demonstrated adhesion defects in a mutant strain lacking the last 20 amino acids of the SipW protein (*SipWΔ170–190*); however, floating biofilm production still showed TasA accumulation at the air–liquid interface, highlighting that this region is not involved in peptidase activity but is involved in adhesion capacity. The same study reports that this region, although not associated with peptidase activity, regulates the expression of other extracellular matrix-related genes in *B. subtilis*, such as the *epsA-O* operon, which contributes to the formation of adhered biofilms on solid surfaces rather than floating biofilms. Whether an analogous regulatory relationship exists in *B. cereus s.l.* remains to be determined, since we did not assess expression of extracellular matrix-associated genes in the present study; this represents a relevant direction for future work in *B. cereus* and *B. pacificus*. This may relate to the biofilm assay performed in this study, which does not specifically assess air–liquid interface biofilms but rather biofilm adhesion to the material used—glass, in this case—stained with safranin. Therefore, these changes in the C-terminal region could be related to modifications in adhesion to the material (Figure 4b).

### 2.5. Structural and Functional Analysis of the TasA Protein

**Figure 5:**
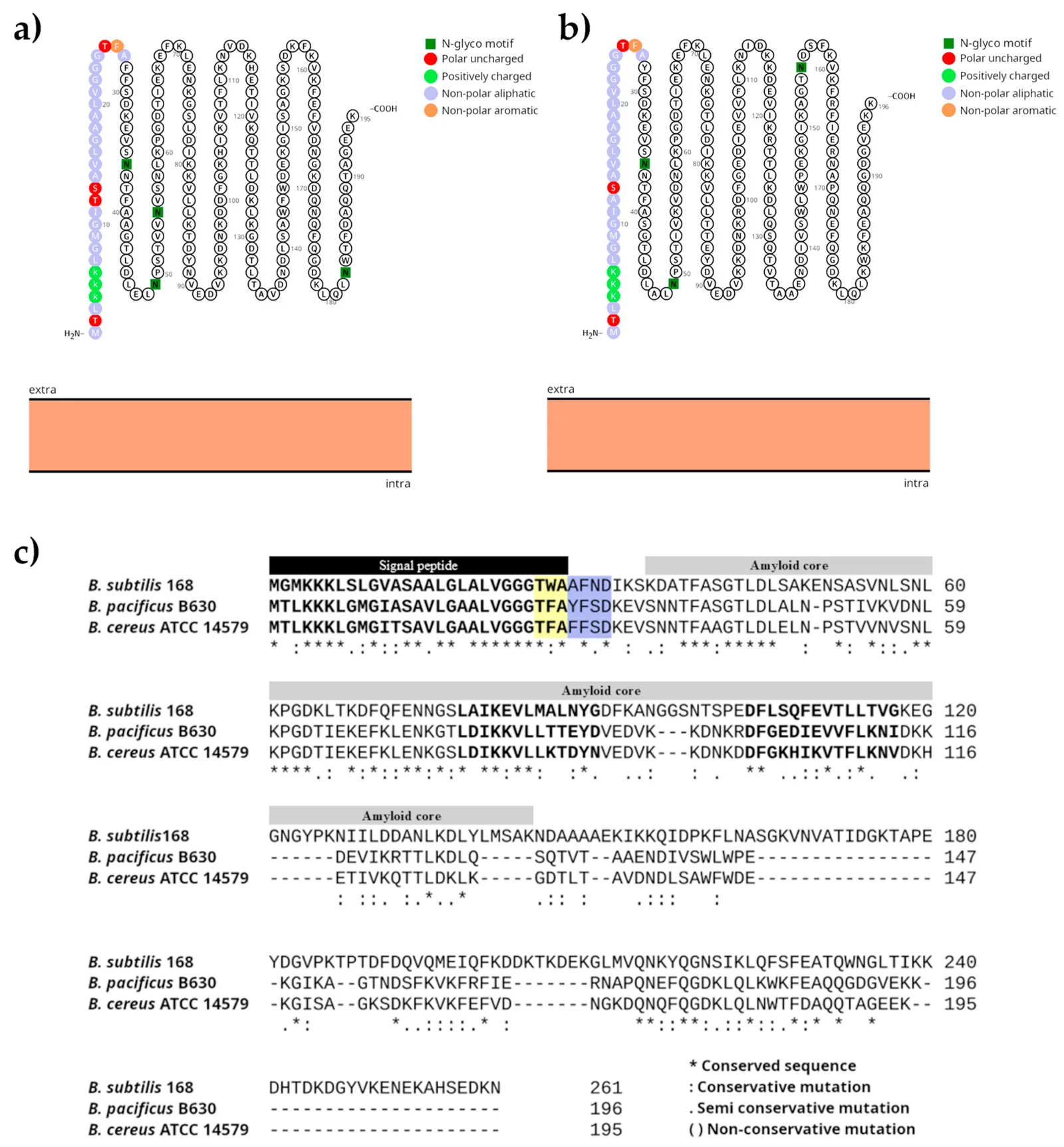
Topological maps of TasA from (**a**) *B. cereus* ATCC 14579 and (**b**) *B. pacificus* B630. The figure was rendered using the Protter web service and edited with GIMP v.3.0. (**c**) Amino acid sequence analysis of immature TasA from *B. pacificus* B630 compared with *B. cereus* ATCC 14579. The Thr–Trp–Ala domain is marked in yellow and the Ala–Phe–Asn–Asp domain in purple.

In *B. subtilis*, the TasA protein is part of the *tapA*-*sipW*-*tasA* gene cluster, whose chromosomal organization and expression pattern indicate that it functions as an operon. TasA is initially synthesized as a preprotein containing a signal peptide located at the N-terminal end, approximately 27 amino acids in length [32,33]. The protein is recognized by the SipW signal peptidase, which removes the signal peptide, enabling secretion and formation of the mature TasA protein, approximately 30 kDa [30]. Once secreted into the extracellular medium, this protein is incorporated into the extracellular matrix that forms part of *B. subtilis* biofilms [25]. The presence of this signal peptide in the amino acid sequences of the immature TasA proteins of *B. cereus* ATCC 14579 and *B. pacificus* B630 was confirmed by analysis with Protter 1.0. This analysis identified the signal peptide in both proteins, 27 amino acids in length, confirming what was previously described by Stöver & Driks (1999) [32]. In this region, a triple lysine motif was identified at positions 4–6, followed by a hydrophobic region composed of nonpolar amino acids, features previously described in signal peptides associated with secretion pathways [30] (Figure 5a).

It has also been described that within the signal peptide there is a cleavage site compatible with signal peptidase [30]. On this point, Musik et al. (2021) [36] demonstrated that the experimentally determined cleavage site occurs between residues Ala27 and Ala28, and that the region surrounding the processing site corresponds to the motif Thr–Trp–Ala│Ala–Phe–Asn–Asp (TWA│AFND) In this study, comparative amino acid sequence analysis of the TasA protein from *B. subtilis* 168, *B. cereus* ATCC 14579, and *B. pacificus* B630 shows that the double alanine within the Thr–Trp–Ala│Ala–Phe–Asn–Asp motif, present in the *B. subtilis* signal peptide, is not conserved in *B. cereus* ATCC 14579 or *B. pacificus* B630; instead, only the first alanine and a subset of flanking residues (TxAxFxD) are retained. Because the double-alanine motif is absent in both strains under study, the exact cleavage site of TasA in *B. cereus* ATCC 14579 and *B. pacificus* B630 cannot be inferred from sequence homology alone and remains to be experimentally determined. It is worth noting that this variation is observed not only relative to *B. subtilis*, but also between *B. cereus* ATCC 14579 and *B. pacificus* B630 themselves, despite both belonging to the same *B. cereus s.l.* group (Figure 5c). Determining the actual processing site in these strains, whether through molecular dynamics simulations of the signal peptide-SipW interaction or through experimental approaches analogous to those used by Musik et al. (2021) [36], represents a relevant direction for future work (Figure 5c).

**Figure 6:**
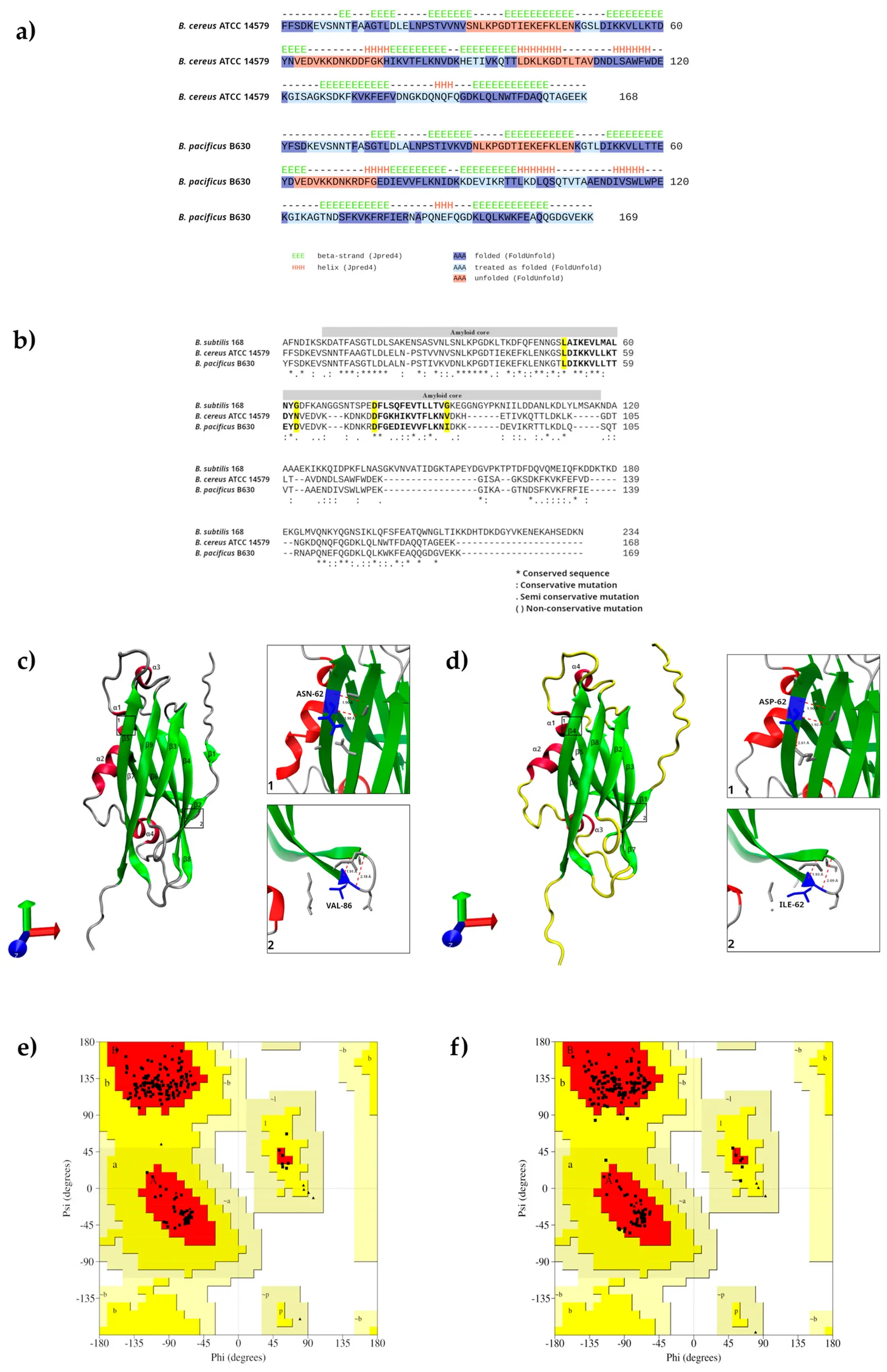
TasA protein of both study strains. (**a**) JPred3 and FoldUnfold were applied to predict secondary structures and disordered regions of TasA in the study strains. (**b**) Amino acid sequence analysis of TasA from *B. pacificus* B630 compared with *B. cereus* ATCC 14579 and *B. subtilis* 168. Amino acids forming part of the high aggregation propensity segments are marked in yellow. (**c**,**d**) AlphaFold3 prediction of the TasA proteins of *B. cereus* ATCC 14579 and *B. pacificus* B630. The yellow model corresponds to *B. pacificus* B630, while the silver model corresponds to *B. cereus* ATCC 14579. Beta sheets are shown in green, and alpha helices in red. The amino acid substitution within the model is indicated in blue. For each prediction, a magnified view of the region containing high aggregation propensity amino acid substitutions is shown on the right. Editing was performed in Visual Molecular Dynamics (VMD) v.1.9.4. Detailed analysis of amino acid interactions was performed in ChimeraX v1.12. (**e**,**f**) Validation model using SAVES (v.6.0) with PROCHECK. After refinement with GalaxyWEB, the Ramachandran plots show that 98.7% of the TasA amino acid residues of *B. cereus* ATCC 14579 and *B. pacificus* B630 are located in the most favored region. Confidence metrics obtained for the structural models of the TasA protein are included in Table S6.

After cleavage of the signal peptide by SipW and its secretion into the extracellular medium, TasA can initially adopt a stable globular conformation before rearranging to form amyloid fibers [27]. The mature TasA protein in *B. subtilis* displays a hybrid organization in which β-sheet-rich regions coexist with α-helical elements [28]. The crystallographic structure of the TasA monomer revealed the presence of type II polyproline helices (PPII), which possess high conformational plasticity, as they can readily convert into α-helices and β-sheets through minimal changes in the torsion angles of amino acid residues. The presence of these PPII helices and 3_10_ helices allows the protein to adopt a structural organization that provides the plasticity required to undergo the conformational changes that give rise to the characteristic cross-β architecture of structural amyloid fibers [27]. In this study, crystallographic data for the TasA proteins of *B. cereus* ATCC 14579 or *B. pacificus* B630 were not available; nevertheless, amino acid sequence analysis allowed the following observations: β-sheet regions could be identified in both proteins, and as a first difference, an additional β-sheet was predicted in *B. cereus* ATCC 14579. In both cases, four α-helices were predicted, two of which correspond to 3_10_ helices. All the previously mentioned secondary structures collectively conform to the general characteristics of an amyloid protein with a jelly-roll fold (Figure 6a). In *B. subtilis*, the stability of this fold depends on an internal β-strand, located between residues 48 and 61, which protects the hydrophobic groove of the protein core. During conformational rearrangement, this β-strand ceases to internally stabilize the protein and is displaced by a new N-terminal β-strand, comprising residues 28–38 of the adjacent subunit, a mechanism termed β-strand exchange [24]. In this study, β-sheets β5 and β4 occupy these positions in *B. cereus* ATCC 14579, and β-sheets β4 and β3 in *B. pacificus* B630. Therefore, the additional β-sheet in *B. cereus* ATCC 14579 is not directly related to β-strand exchange. However, it is worth noting that the amino acids composing these β-sheets differ in primary sequence between TasA of both strains. Future molecular dynamics simulations of TasA polymerization between these strains could help clarify the impact of these amino acid changes on β-sheet interactions.

In *B. subtilis*, the rigid N-terminal region, comprising residues K35–K144, has been described as constituting the structural core responsible for the amyloid properties of the protein. In this study, changes were identified at both residues: at position K35, a substitution to S35 was found in both strains; at position K144, this corresponds to K129 in *B. cereus* ATCC 14579 and Q129 in *B. pacificus* B630. In addition to the variability in the rigid N-terminal region, alignment of the amino acid sequences of the immature protein from both study strains reveals high variability relative to *B. subtilis* 168, and variability is even predicted between the two study strains. Although the similarity between TasA of *B. subtilis* and *B. cereus* has been reported as 35%, both proteins retain the ability to assemble into functional fibers, which is related to preservation of their three-dimensional structural organization rather than similarity of their primary sequence [28]. In this regard, it has been described that within the amyloid core of the *B. subtilis* sequence, two segments with high aggregation propensity are found, designated LG-13 (Leu78–Gly90) and DG-14 (Asp104–Gly117) [26]. Upon identifying the location of these residues in the TasA sequences of *B. cereus* ATCC 14579 and *B. pacificus* B630, it was found that only Leu78 and Asp104 are conserved, with differences at Gly90 and Gly117; these differences are predicted not only relative to *B. subtilis* 168, but also between the sequences of the two study strains (Figure 6b). The presence of these residues, together with a series of amino acid repeats, favors the transition toward β-sheet-rich structures and constitutes the molecular basis for the formation of fibers capable of mechanically supporting the extracellular matrix [26]. Figure 6c,d show that, although the substitution of asparagine with aspartic acid (N90D) and valine with isoleucine (V117I) represent conserved amino acid replacements, these changes are predicted to alter local electrostatic properties, which could in principle affect hydrogen bond formation, ionic interactions, and other local contacts. However, these observations are based on static monomeric models and do not capture the conformational rearrangements and donor-strand exchange that TasA undergoes during fiber polymerization; therefore, their functional impact on biofilm formation cannot be inferred from the present structural predictions alone. Molecular dynamics simulations of TasA oligomers, or experimental fiber aggregation assays, would be required to determine whether these substitutions actually influence amyloid assembly in the studied strains.

## 3. Materials and Methods

### 3.1. Strain Reactivation

In this study, *B. cereus* ATCC 14579 and *B. pacificus* B630 were used. The latter was isolated from rice samples using mannitol egg yolk polymyxin agar and initially classified within the *B. cereus s.l.* group by amplification of the *gyrB* gene [12]. Subsequently, its taxonomic identity as *B. pacificus* was established through phylogenomic analysis [13].

Both strains are preserved at −20 °C in the biobank of the Laboratorio de Investigación en Patometabolismo Microbiano (Microbial Pathometabolism Research Laboratory). For reactivation, each strain was inoculated into BHI broth (Brain Heart Infusion; BD Bioxon) and incubated at 30 °C for 24 h.

### 3.2. Biofilm Production

Biofilm formation was assessed in glass tubes containing 1 mL of BHI broth (Brain Heart Infusion; BD Bioxon, Franklin Lakes, NJ, USA) supplemented with 1% (*w*/*v*) dextrose. Each tube was inoculated with a 24-h liquid culture of *B. cereus* ATCC 14579 or *B. pacificus* B630, adjusted to a concentration of approximately 6 × 10^6^ CFU/mL. Tubes were then incubated at 30 °C for 48 h under static conditions to promote biofilm formation.

After the incubation period, the bacterial culture was carefully removed and the tubes were washed three times with phosphate-buffered saline (1X PBS; 137 mM NaCl, 2.7 mM KCl, 10 mM Na_2_HPO_4_, 1.8 mM KH_2_PO_4_, pH 7.2) (Life Technologies, Carlsbad, CA, USA) to remove non-adherent cells. Biofilms formed on the inner surface of the tubes were stained with 0.1% safranin solution (BD Difco, Franklin Lakes, NJ, USA) for 60 min under constant agitation. Tubes were then washed three times with 1X PBS, and the safranin retained in the biofilm was solubilized by adding absolute ethanol (Wöhler, Iztapalapa, Mexico City, Mexico) for 30 min. Finally, the amount of biofilm formed was determined by measuring the absorbance of the solubilized safranin solution at 590 nm [15].

### 3.3. Motility Assay

Motility was determined in a semisolid medium consisting of BHI broth supplemented with 0.5% (*w*/*v*) bacteriological agar. The medium was poured into sterile Petri dishes and allowed to solidify before inoculation. An isolated colony of *B. cereus* ATCC 14579 or *B. pacificus* B630 was then inoculated at the center of each plate. Plates were incubated at 30 °C for 24 h. After incubation, colony diameter was measured with a caliper as an indicator of bacterial motility. *B. cereus* ATCC 14579 was used as a control for comparison with *B. pacificus* B630. All assays were performed in triplicate across two independent experiments [15].

### 3.4. Statistical Analysis

Data from biofilm production and motility assays were analyzed using statistical tests that allow comparison between two groups—in this case, *B. cereus* ATCC 14579 and *B. pacificus* B630. Results are expressed as the mean and standard deviation, reflecting data obtained from triplicate measurements across two independent experiments.

To compare results between strains, we used an independent-samples Student’s *t*-test. In all analyses, a *p*-value < 0.05 was considered statistically significant.

Data obtained from the biofilm production and motility assays were graphically represented using GraphPad Prism v. 8.0.2 software (GraphPad Software, San Diego, CA, USA). Statistical analyses were performed using Stata v. 16.0 software (StataCorp LLC, College Station, TX, USA).

### 3.5. Orthologous Cluster Analysis 

For the comparative analysis of orthologous gene families, the genomes of *B. cereus* ATCC 14579 (accession GCA_046524075.1) and *B. pacificus* B630 (accession GCA_051917385.1). Both were retrieved from the GenBank database (https://www.ncbi.nlm.nih.gov/genbank/, accessed on 14 July 2026). Based on data from this database, we verified the assembly quality of both genomes: they have similar sizes and GC content, high sequencing coverage, and high completeness according to CheckM (98.59% for *B. cereus* ATCC14579 and 98.82% for *B. pacificus* B630), with minimal contamination. Furthermore, we annotated both RefSeq genomes using the NCBI Prokaryotic Genome Annotation Pipeline (PGAP) v. 6.11, using successive versions of the pipeline. We used the *.faa output files from the annotation as input for the OrthoVenn3 web server (https://orthovenn3.bioinfotoolkits.net/, accessed on 15 July 2026).

Orthology analysis was performed using the OrthoFinder algorithm implemented in OrthoVenn3 to identify clusters of orthologous proteins. An e-value cutoff of 1 × 10^−2^ was used for protein alignments, and an inflation value (I) of 1.5 was used for sequence clustering. In addition, the Enable Annotation and Protein Similarity options were activated to incorporate functional annotation and protein similarity analysis during the comparison.

OrthoVenn3 generated a Venn diagram that visualized the distribution of shared and exclusive orthologous protein families between the analyzed strains, as well as the total number of orthologous families identified in each genome. Protein families exclusive to *B. cereus* ATCC 14579 and *B. pacificus* B630 were subsequently analyzed. For each cluster, the number of proteins, the identifier of the homologous protein in the Swiss-Prot database, and the functional annotation were recorded.

Additionally, proteins exclusive to each strain were classified according to Gene Ontology (GO) terms assigned by OrthoVenn3. GO terms were grouped using the corresponding GO Slim classification for the biological process category, recording the number of proteins associated with each process.

### 3.6. Search for Genes Related to Extracellular Matrix Production

A preliminary analysis was carried out using the genomic sequences of *B. cereus* ATCC 14579 (accession GCA_046524075.1) and *B. pacificus* B630 (accession GCA_051917385.1). Both were retrieved from the GenBank database (https://www.ncbi.nlm.nih.gov/genbank/, accessed on 16 July 2026). Identification of genes involved in biofilm synthesis was performed by querying the Virulence Factors of Pathogenic Bacteria database (VFDB; https://www.mgc.ac.cn/VFs/, accessed on 17 July 2026) to obtain the list of genes in the capsular polysaccharide category associated with extracellular matrix formation and biofilm development.

The genes identified in this category were then located in both genomes by reviewing the annotation files (.gff), recording their presence and genomic position. A local database was constructed for each strain and used to perform pairwise comparisons using the BLASTn algorithm, using the corresponding sequences of *B. cereus* ATCC 14579 and *B. pacificus* B630 as reference. The resulting alignments allowed calculation of nucleotide identity percentages between homologous genes of both strains.

Once the genes associated with the capsular polysaccharide category were identified, the annotation files of both strains were loaded into SnapGene v.8.2 software to locate and visualize the genomic organization. Based on the arrangement of these genes, it was established that they corresponded to the *eps1* operon. The genes composing this operon were then delimited according to genomic position and nucleotide sequence and subsequently compared using the BLASTn algorithm to establish correspondence between homologous genes. Based on the concordance between sequence homology and the annotations obtained with both tools, a putative function was assigned to each gene, and differences in functional annotation observed between homologous genes of both strains were recorded.

### 3.7. Identification and Comparative Analysis of the sipW-tasA-calY Operon

Identification and delimitation of the *sipW*-*tasA*-*calY* operon were performed by reviewing the genomic context in the annotation files (.gff) of *B. pacificus* B630 and, *B. cereus* ATCC 14579, from the access codes *B. cereus* ATCC 14579 (access code GCA_046524075.1) and *B. pacificus* B630 (access code GCA_051917385.1), both retrieved from the GenBank database (https://www.ncbi.nlm.nih.gov/genbank/, accessed on 18 July 2026).

Genomic inspection identified the region composed of the *sipW*, *tasA*, *BC_1280*, and *calY* genes, corresponding to the *sipW*-*tasA*-*calY* locus, in accordance with the organization previously described for bacteria of the *B. cereus* group [22]. For visualization and comparison of locus architecture between both strains, the annotation files were imported into SnapGene v.8.2 software, recording gene order, orientation, and spatial arrangement.

Once the locus was delimited, the nucleotide sequences of the *sipW*, *tasA*, *BC_1280*, and *calY* genes were retrieved from the coding sequence (.cds) files corresponding to each genome and organized into independent local databases for *B. cereus* ATCC 14579 and *B. pacificus* B630. Homologous sequences of both strains were then compared pairwise using the BLASTn algorithm to determine the percentage of nucleotide identity for each gene analyzed.

Finally, the schematic representation of the operon obtained with SnapGene was integrated into a single figure using GIMP v.3.0 software.

### 3.8. Prediction of SipW Protein Topology

Once the genes of interest were identified, the amino acid sequence of the SipW protein of *B. pacificus* B630 was retrieved from the protein files (.faa) from the access codes *B. cereus* ATCC 14579 (access code GCA_046524075.1) and *B. pacificus* B630 (access code GCA_051917385.1), both retrieved from the GenBank database (https://www.ncbi.nlm.nih.gov/genbank/, accessed on 19 July 2026).

The sequences of both strains were analyzed using the Protter web server (https://protter.ethz.ch, accessed on 20 July 2026), which employs the Phobius algorithm to predict the topology of membrane proteins. This analysis allowed a topological representation of SipW to be obtained, identifying the location of transmembrane domains as well as the position of amino acid residues located within the membrane. Finally, the topological representations obtained for *B. cereus* ATCC 14579 and *B. pacificus* B630 were integrated using GIPM v.3.0 software.

### 3.9. Modeling, Comparative Analysis, and Structural Validation of the SipW Protein

The amino acid sequences of SipW from *B. pacificus* B630 (GCA_051917385.1), *B. pacificus* ATCC 10987 (GCA_031316815.1), *B. cereus* ATCC 14579 (GCA_046524075.1), and *B. subtilis* 168 (GCA_000009045.1) were retrieved from the corresponding .faa protein files of each genome. *B. subtilis* 168 was included as a reference strain, as the SipW protein has been extensively functionally characterized in this species and constitutes a model for the study of signal peptidases involved in biofilm formation [35].

The amino acid sequences in FASTA format were subjected to structural modeling using the AlphaFold3 web server (https://alphafoldserver.com/, accessed on 21 July 2026), which employs artificial intelligence to predict three-dimensional protein structures from amino acid sequences [37]. A three-dimensional model in Protein Data Bank (.pdb) format was obtained for each protein. The AlphaFold models are available in ModelArchive (https://modelarchive.org) with the accession codes ma-vahlv, ma-whjq4.

The resulting three-dimensional models were visualized with Visual Molecular Dynamics (VMD) v.1.9.4, in which structural alignment was performed by superimposing the α-carbons (Cα) of the SipW proteins from the four strains, using the structural alignment tools available in the software.

The stereochemical quality of the resulting models was assessed using the PROCHECK tool, available on the Structural Analysis and Verification Server (SAVES v.6.1; https://saves.mbi.ucla.edu/, accessed on 22 July 2026). A Ramachandran plot was generated for each model, and the evaluation criterion considered was the percentage of amino acid residues located in the most favored, additionally allowed, generously allowed, and disallowed regions.

Because the initial models showed percentages of residues in the most favored regions below those recommended for high-quality structural models, they were subjected to a refinement process using the GalaxyWeb server (https://galaxy.seoklab.org/, accessed on 23 July 2026) and the GalaxyRefine tool, designed to improve the structural quality of protein models through side-chain refinement and structural relaxation [38]. The refined models were then re-evaluated using PROCHECK on the SAVES v.6.1 server, applying the same stereochemical quality criteria for generating Ramachandran plots and selecting the final models.

Finally, the structural alignment representation obtained in VMD and the Ramachandran plots generated with PROCHECK were integrated into a single figure using GIMP v.3.0 software.

### 3.10. Structural and Functional Characterization of the SipW Protein

The previously retrieved amino acid sequences of SipW from *B. pacificus* B630 and *B. cereus* ATCC 14579 were aligned using the Clustal Omega web server (https://www.ebi.ac.uk/jdispatcher/msa/clustalo, accessed on 24 July 2026) to compare the conservation of amino acid residues between the two proteins.

Sequences were subsequently analyzed using the PROSITE web server of ExPASy (https://prosite.expasy.org/, accessed on 25 July 2026) to identify conserved motifs and residues associated with the catalytic activity of the SipW protein. Assignment of the active site and the residues forming the catalytic triad was based on information reported for the SipW protein of *B. subtilis* 168 by Tjalsma et al. (2000) [34], comprising serine (Ser), histidine (His), and aspartic acid (Asp), characteristic of type I signal peptidases.

The amino acid alignment obtained with Clustal Omega was combined with the previously obtained transmembrane domain localization data from Protter, as well as the location of the active site and the residues forming the catalytic triad. Editing of the alignment was performed using GIMP v.3.0.

The previously obtained three-dimensional models were edited in VMD v.1.9.4 to highlight the location of the active site and the residues forming the catalytic triad, using a consistent color code between both proteins. The Ramachandran plots obtained during stereochemical validation of the models with PROCHECK on the SAVES v.6.1 server were also incorporated. Finally, panel integration and editing were performed using GIMP v.3.0 software.

### 3.11. Identification of the Signal Peptide and Functional Regions of TasA

Following characterization of the SipW protein, the previously retrieved amino acid sequences of TasA from *B. pacificus* B630 and *B. cereus* ATCC 14579 were analyzed. Sequences were analyzed using the Protter web server (https://protter.ethz.ch/start/, accessed on 26 July 2026) to identify the signal peptide located at the N-terminal end of the protein, corresponding to the first 27 amino acid residues, as previously described for the TasA protein of *B. subtilis* [27].

Subsequently, the homologous TasA sequence of *B. subtilis* 168, obtained from the reference genome (accession GCA_000009045.1), was incorporated, as the structural organization and functional regions of this protein have been experimentally characterized in this species [26,27]. Sequences of the three strains were aligned using the Clustal Omega web server to compare amino acid sequence conservation and identify the functional regions described for the TasA protein.

From the alignment, the signal peptide, the central amyloidogenic core region, the two regions with amyloidogenic potential (amyloidogenic stretches I and II), and differences in length and amino acid variation between the proteins were identified, based on the functional regions described by Cámara-Almirón et al. (2023) [26]. Finally, the amino acid alignment was edited with GIMP v.3.0 software, highlighting the signal peptide and functional regions.

### 3.12. Structural and Functional Characterization of the TasA Protein

To characterize the structural properties of TasA, the amino acid sequences of *B. pacificus* B630 and *B. cereus* ATCC 14579 were analyzed using the JPred3 web server (http://www.compbio.dundee.ac.uk/jpred/, accessed on 27 July 2026) for secondary structure prediction, to identify the distribution of α-helices, 3_10_ helices, and β-sheets. The same sequences were additionally analyzed using the FoldUnfold server (http://bioinfo.protres.ru/ogu/, accessed on 28 July 2026) to predict structurally ordered and disordered regions.

Subsequently, the amino acid sequences of TasA from *B. pacificus* B630 and *B. cereus* ATCC 14579 were aligned with the homologous sequence of *Bacillus subtilis* 168 using Clustal Omega to compare the conservation of amino acid residues and identify the regions with high aggregation propensity previously described for this protein. The LG-13 (Leu78–Gly90) and DG-14 (Asp104–Gly117) segments were used as reference, as well as the amino acid substitutions present between the proteins, according to Cámara-Almirón et al. (2023) [26]. Editing of the alignment and highlighting of the regions of interest were performed using GIMP v.3.0 software.

Based on the mature form of TasA, corresponding to the protein after signal peptide processing, three-dimensional models were generated using the AlphaFold3 web server following the same methodological strategy previously described for SipW. The resulting models were subjected to structural refinement using GalaxyWeb and subsequently validated with the PROCHECK tool on the SAVES v.6.1 server, through generation of Ramachandran plots to assess their stereochemical quality. The AlphaFold models are available in ModelArchive (https://modelarchive.org) with the accession codes ma-uxigt and ma-6ayk3.

The refined three-dimensional models were analyzed with VMD v.1.9.4 software, in which the predicted secondary structure elements for each protein, including α-helices and β-sheets, were identified and colored. The models were then analyzed in ChimeraX v1.12, where magnifications of the regions with high aggregation propensity (LG-13 and DG-14) were generated to examine the amino acid substitutions present between the proteins of *B. pacificus* B630 and *B. cereus* ATCC 14579, and their possible influence on the local conformation of these regions.

Finally, the secondary structure predictions obtained with JPred3 and FoldUnfold, the amino acid alignment, the three-dimensional models, the magnified views of the regions of interest, and the Ramachandran plots were integrated into a single figure using GIMP v.3.0 software.

## 4. Conclusions

Biofilm production has been extensively studied in *B. subtilis*, whereas less information is available for *B. cereus*. This study identified differences in biofilm production and motility between the strains *B. cereus* ATCC 14579 and *B. pacificus* B630, both belonging to the *B. cereus s.l.* group. Differences were also found at the genomic and molecular prediction levels; these warrant further exploration at the transcriptomic and proteomic levels, which would provide new insights into the molecular mechanisms of biofilm production in these strains.

## Figures and Tables

**Table 1:** Nucleotide identity percentage of homologous genes of the *eps1* and *sipW*-*tasA*-*calY* regions of *B. pacificus* B630 compared with *B. cereus* ATCC 14579.

	*B. pacificus* B630
*eps1* region	
*BC5263*	93.95%
*BC5264*	87.60%
*BC5265*	84.64%
*BC5274*	83.99%
*BC5275*	93.19%
*BC5276*	86.97%
*BC5277*	90.25%
*BC5278*	87.68%
*BC5279*	90.72%
*sipW-tasA-calY* region	
*sipW*	89.14%
*tasA*	78.43%
*Bc_1280*	71.29%
*calY*	84.51%

Comparison of the operons using the BLASTn algorithm and coverage executed in Linux to determine nucleotide sequence identity percentages.

## Data Availability

The original contributions presented in this study are included in the article and Supplementary Materials. Further inquiries can be directed to the corresponding authors.

## Supplementary Materials

The following supporting information can be downloaded at: https://www.mdpi.com/article/10.3390/ijms27177743/s1.
