# Supplementary material for: Bioinformatic Analysis of Bacillus pacificus B630: Molecular Understanding of Biofilm Production
Source: Int J Mol Sci. 2026 Aug 29;27(17):7743. doi: 10.3390/ijms27177743 (PMC13566338; doi:10.3390/ijms27177743)
Supplement: Supplementary file 1 [file ijms-27-07743-s001.zip › ijms-4501333-supplementary.pdf]

**Table S1.** List of proteins on *B. pacificus* B630

| Cluster ID | Number of proteins | Swiss-Pro Hit | Annotation |
|---|---|---|---|
| Cluster 30 | 7 | P54992 | GO:0032196; P:transposition; IEA:UniProtKB-KW |
| Cluster 31 | 7 | P06693 | GO:0015074; P:DNA integration; IEA:UniProtKB-KW |
| Cluster 32 | 7 | Q47688 | N/A |
| Cluster 97 | 5 | N/A | N/A |
| Cluster 98 | 5 | Q07833 | GO:0016078; P:tRNA catabolic process; IDA:UniProtKB |
| Cluster 269 | 4 | P96649 | GO:0004721; F:phosphoprotein phosphatase activity; IEA:UniProtKB-KW |
| Cluster 270 | 4 | N/A | N/A |
| Cluster 271 | 4 | N/A | N/A |
| Cluster 272 | 4 | P68579 | GO:0015031; P:protein transport; IEA:UniProtKB-KW |
| Cluster 273 | 4 | P46352 | GO:0006313; P:transposition, DNA-mediated; IEA:UniProtKB-UniRule |
| Cluster 274 | 4 | N/A | N/A |
| Cluster 606 | 3 | O25526 | GO:0071555; P:cell wall organization; IEA:UniProtKB-KW |
| Cluster 607 | 3 | P10021 | GO:0006313; P:transposition, DNA-mediated; IEA:InterPro |
| Cluster 608 | 3 | N/A | N/A |
| Cluster 609 | 3 | P13991 | GO:0032196; P:transposition; IEA:UniProtKB-KW |
| Cluster 610 | 3 | N/A | N/A |
| Cluster 4233 | 2 | P05846 | GO:0032196; P:transposition; IEA:UniProtKB-KW |
| Cluster 4234 | 2 | N/A | N/A |
| Cluster 4235 | 2 | N/A | N/A |
| Cluster 4236 | 2 | P54549 | N/A |
| Cluster 4237 | 2 | N/A | N/A |
| Cluster 4238 | 2 | Q82ZG8 | GO:0046872; F:metal ion binding; IEA:UniProtKB-KW |
| Cluster 4239 | 2 | O31990 | GO:0006281; P:DNA repair; IEA:UniProtKB-KW |
| Cluster 4240 | 2 | N/A | N/A |
| Cluster 4241 | 2 | N/A | N/A |
| Cluster 4242 | 2 | N/A | N/A |
| Cluster 4243 | 2 | N/A | N/A |
| Cluster 4244 | 2 | P13989 | GO:0006313; P:transposition, DNA-mediated; IDA:UniProtKB |
| Cluster 4245 | 2 | P49156 | GO:0043640; P:benzoate catabolic process via hydroxylation; IEA:UniProtKB-UniPathway |
| Cluster 4246 | 2 | N/A | N/A |
| Cluster 4247 | 2 | Q9ZFB3 | GO:0009847; P:spore germination; IEA:InterPro |
| Cluster 4248 | 2 | N/A | N/A |
| Cluster 4249 | 2 | Q8Z8T1 | GO:0052689; F:carboxylic ester hydrolase activity; IEA:UniProtKB-UniRule |
| Cluster 4250 | 2 | N/A | N/A |

| | | | |
|---|---|---|---|
| Cluster 4251 | 2 | N/A | N/A |
| Cluster 4252 | 2 | N/A | N/A |
| Cluster 4253 | 2 | N/A | N/A |
| Cluster 4254 | 2 | N/A | N/A |
| Cluster 4255 | 2 | P13988 | GO:0006313; P:transposition, DNA-mediated; IDA:UniProtKB |
| Cluster 4256 | 2 | A4IT51 | GO:0019439; P:aromatic compound catabolic process; IEA:UniProtKB-KW |
| Cluster 4257 | 2 | N/A | N/A |
| Cluster 4258 | 2 | N/A | N/A |
| Cluster 4259 | 2 | N/A | N/A |
| Cluster 4260 | 2 | N/A | N/A |
| Cluster 4261 | 2 | N/A | N/A |

**Table S2.** List of proteins and biological processes on *B. pacificus* B630

| Slimmed GO | Count of unique input accessions | Name |
|---|---|---|
| GO:0008152 | 15 | metabolic process |
| GO:0044237 | 13 | cellular metabolic process |
| GO:0043170 | 11 | macromolecule metabolic process |
| GO:0006725 | 9 | cellular aromatic compound metabolic process |
| GO:0032196 | 9 | transposition |
| GO:0006139 | 8 | nucleobase-containing compound metabolic process |
| GO:0006807 | 8 | nitrogen compound metabolic process |
| GO:0008150 | 8 | biological_process |
| GO:0044238 | 8 | primary metabolic process |
| GO:0046483 | 8 | heterocycle metabolic process |
| GO:0006259 | 6 | DNA metabolic process |
| GO:0016070 | 4 | RNA metabolic process |
| GO:0006082 | 3 | organic acid metabolic process |
| GO:0015074 | 3 | DNA integration |
| GO:0006396 | 2 | RNA processing |
| GO:0005975 | 1 | carbohydrate metabolic process |
| GO:0005976 | 1 | polysaccharide metabolic process |
| GO:0006281 | 1 | DNA repair |
| GO:0009987 | 1 | cellular process |
| GO:0015031 | 1 | protein transport |
| GO:0017144 | 1 | drug metabolic process |
| GO:0032502 | 1 | developmental process |
| GO:0071555 | 1 | cell wall organization |

**Table S3.** List of proteins on *B. cereus* ATCC 14579

| Cluster ID | Number of proteins | Swiss-Pro Hit | Annotation |
|---|---|---|---|
| Cluster55 | 5 | N/A | N/A |
| Cluster57 | 5 | P96649 | GO:0004721; F:phosphoprotein phosphatase activity; IEA:UniProtKB-KW |
| Cluster313 | 3 | N/A | N/A |
| Cluster348 | 3 | N/A | N/A |
| Cluster382 | 3 | N/A | N/A |
| Cluster436 | 3 | P80172 | GO:0044179; P:hemolysis in other organism; IEA:UniProtKB-KW |
| Cluster535 | 3 | N/A | N/A |
| Cluster620 | 2 | N/A | N/A |
| Cluster821 | 2 | N/A | N/A |
| Cluster1081 | 2 | N/A | N/A |
| Cluster1259 | 2 | N/A | N/A |
| Cluster1368 | 2 | P42179 | GO:0043565; F:sequence-specific DNA binding; IEA:InterPro |
| Cluster1567 | 2 | C0H3X4 | N/A |
| Cluster1788 | 2 | N/A | N/A |
| Cluster1834 | 2 | N/A | N/A |
| Cluster1900 | 2 | N/A | N/A |
| Cluster1980 | 2 | N/A | N/A |
| Cluster2523 | 2 | N/A | N/A |
| Cluster2603 | 2 | N/A | N/A |
| Cluster2748 | 2 | N/A | N/A |
| Cluster3028 | 2 | N/A | N/A |
| Cluster3059 | 2 | Q813A2 | N/A |
| Cluster3101 | 2 | A0A0H2XI99 | GO:0009405; P:pathogenesis; IEA:UniProtKB-KW |
| Cluster3452 | 2 | Q9AHT6 | GO:0009986; C:cell surface; IEA:UniProtKB-SubCell |
| Cluster3648 | 2 | N/A | N/A |
| Cluster3720 | 2 | Q47318 | GO:0019290; P:siderophore biosynthetic process; IEA:InterPro |
| Cluster3824 | 2 | N/A | N/A |

**Table S4.** List of proteins and biological processes on *B. cereus* ATCC 14579

| Slimmed GO | Count of unique input accessions | Name |
|---|---|---|
| GO:0008150 | 5 | biological_process |
| GO:0009987 | 4 | cellular process |
| GO:0006518 | 2 | peptide metabolic process |
| GO:0006807 | 2 | nitrogen compound metabolic process |
| GO:0008152 | 2 | metabolic process |
| GO:0019748 | 2 | secondary metabolic process |
| GO:0043603 | 2 | cellular amide metabolic process |
| GO:0044237 | 2 | cellular metabolic process |
| GO:0051186 | 2 | cofactor metabolic process |
| GO:0051704 | 2 | multi-organism process |
| GO:0001906 | 1 | cell killing |

| | | |
|---|---|---|
| GO:0006066 | 1 | alcohol metabolic process |
| GO:0006082 | 1 | organic acid metabolic process |
| GO:0006725 | 1 | cellular aromatic compound metabolic process |
| GO:0031640 | 1 | killing of cells of other organism |
| GO:0032502 | 1 | developmental process |
| GO:0065007 | 1 | biological regulation |

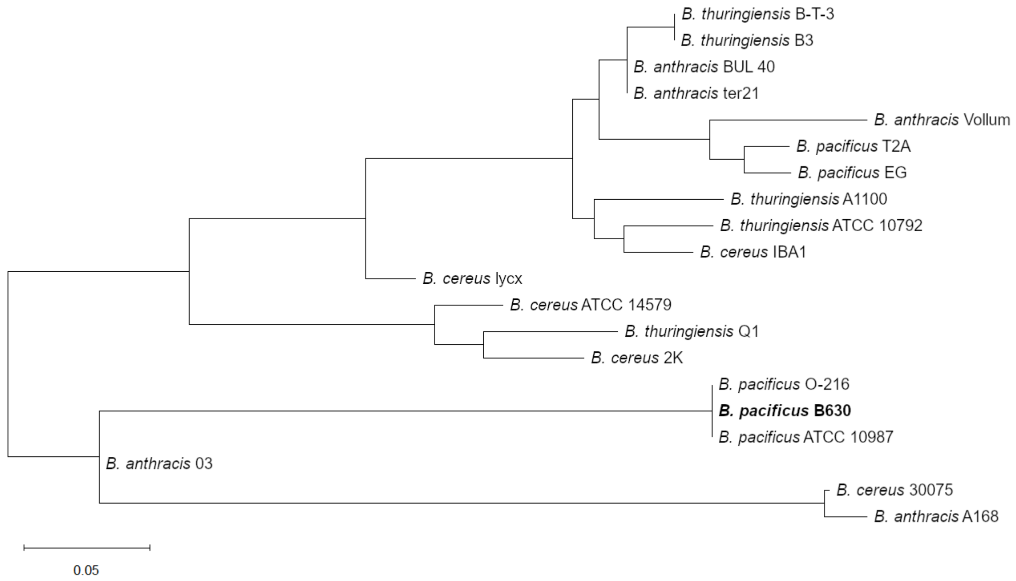


**Figure S1.** Phylogenetic tree from the *eps1* region of *B. cereus* s.l. strains. including *B. cereus* ATCC14579 and *B. pacificus* B630. The tree was constructed using the maximum likelihood method; branch support was assessed using 1,000 bootstrap replicates. The genetic distances were calculated using the p-distance method. The optimal tree is shown.

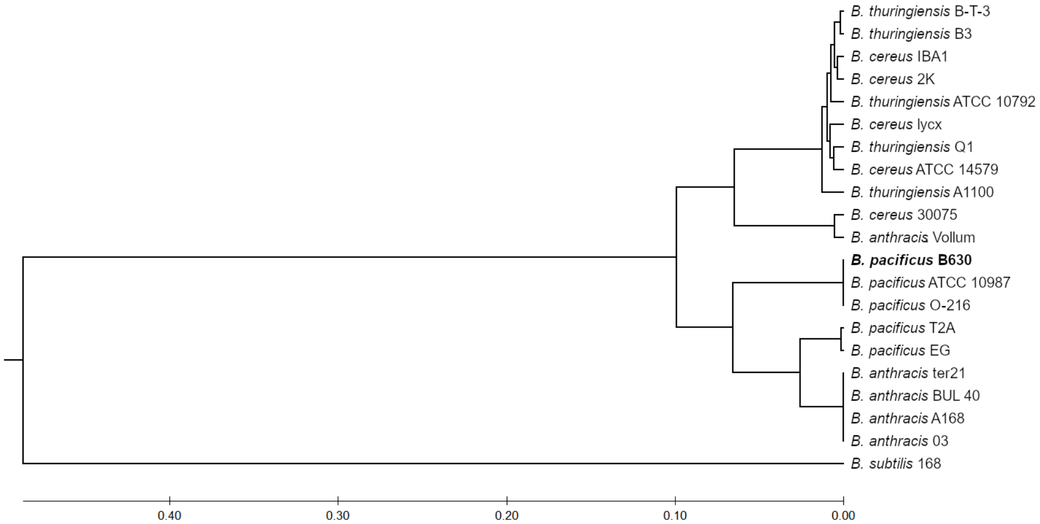


**Figure S2.** Phylogenetic tree from the *sipW- tasA- calY* region of *B. cereus* s.l. strains. including *B. cereus* ATCC14579 and *B. pacificus* B630. We constructed the tree using the UPGMA method; branch support was assessed using 1,000 bootstrap replicates. The genetic distances were calculated using the p-distance method. The optimal tree is shown.

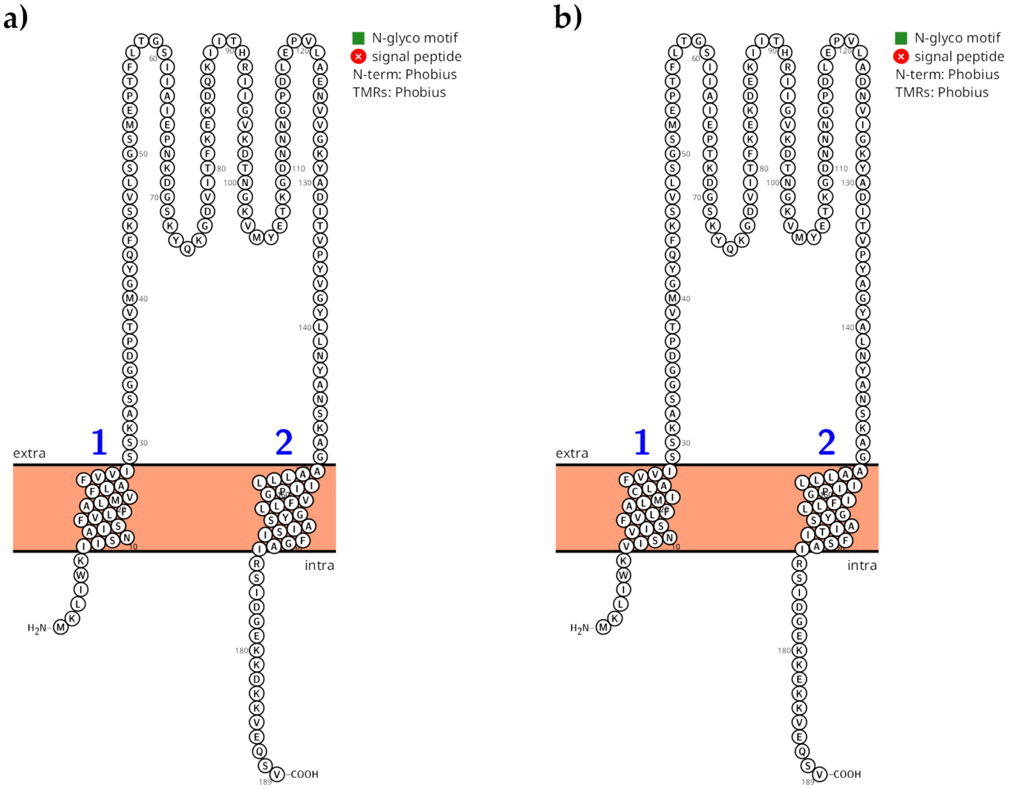


**Figure S3.** Topological maps of SipW from (a) *B. cereus* ATCC 14579 and (b) *B. pacificus* B630.* The figure was rendered using the Protter web service.

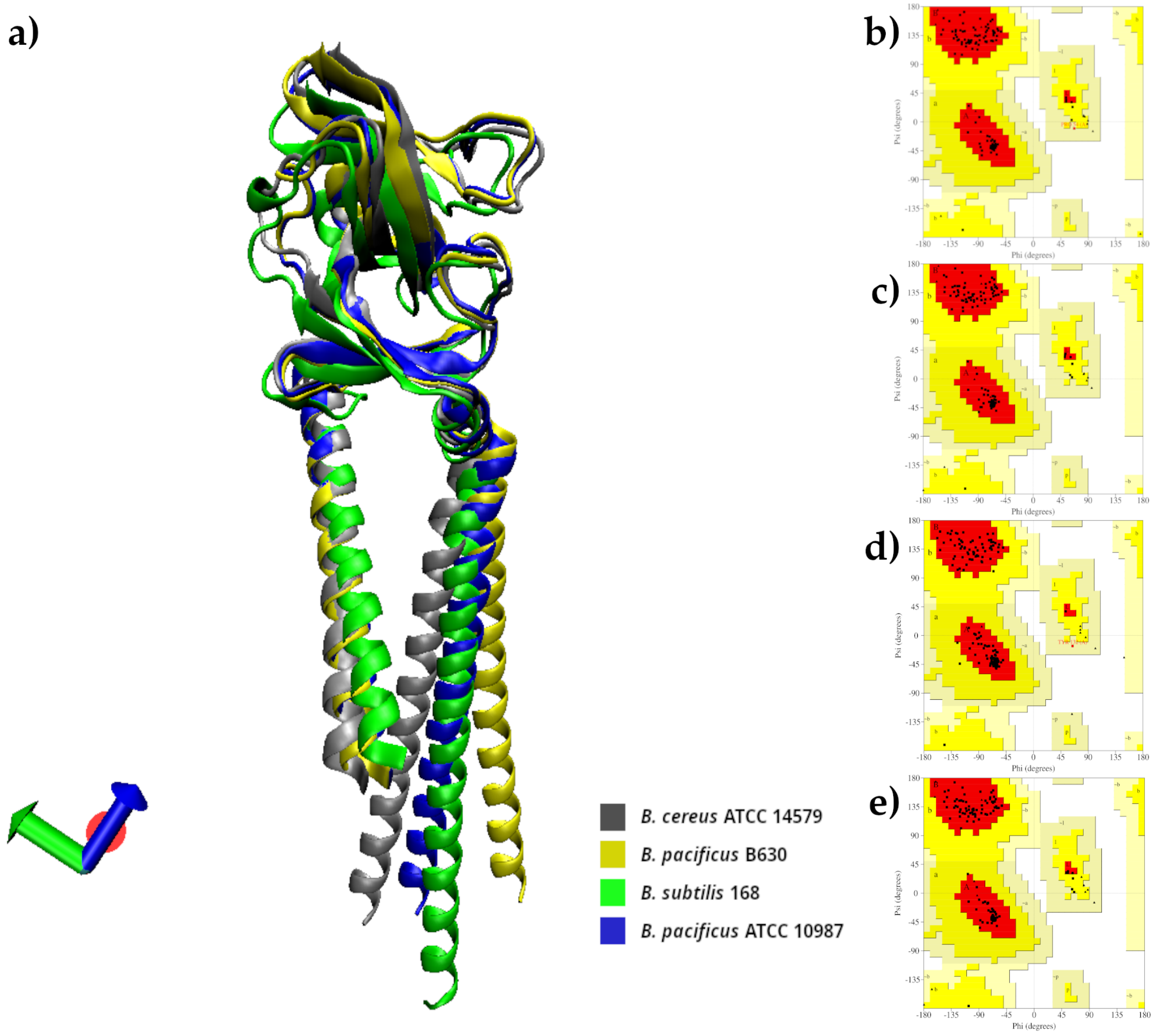


**Figure S4.** (a) AlphaFold3 prediction of the SipW proteins from different *Bacillus* strains. Editing was performed in Visual Molecular Dynamics (VMD) v.1.9.4. (b–e) Validation model using the Structural Analysis and Verification Server (SAVES v.6.1) with the PROCHECK tool. After refinement with GalaxyWEB, the Ramachandran plots show that 98.1%, 98.2%, 97.6%, and 98.2% of the SipW amino acid residues of *B. cereus* ATCC 14579, *B. pacificus* B630, *B. subtilis* 168, and *B. pacificus* ATCC 10987, respectively, are located in the most favored region.

**Tabla S5.** Confidence metrics obtained for the structural models of the SipW protein

| Strain | plDD | PAE (Å) | pTM | ipTM |
|---|---|---|---|---|
| *B. cereus* ATCC 14579 | 77.88 | 9.40 | 0.66 | N/A |
| *B. pacificus* B630 | 84.38 | 7.94 | 0.70 | N/A |

Note: pLDDT is the average per atom. The average PAE was calculated between residue pairs, excluding the main diagonal. N/A: not applicable.

**Table S6.** Confidence metrics obtained for the structural models of the mature TasA protein

| Cepa | plDD | PAE (Å) | pTM | ipTM |
|---|---|---|---|---|
| *B. cereus* ATCC 14579 | 80.55 | 6.82 | 0.81 | N/A |
| *B. pacificus* B630 | 79.53 | 7.34 | 0.79 | N/A |

Note: pLDDT is the average per atom. The average PAE was calculated between residue pairs, excluding the main diagonal. N/A: not applicable.
